# Progressive Sensorineural Hearing Loss in Vibrant Soundbridge Users Requiring Cochlear Implantation

**DOI:** 10.3390/jpm12020191

**Published:** 2022-01-31

**Authors:** Faris F. Brkic, Wolf-Dieter Baumgartner, Dominik Riss, Thomas Thurner, David T. Liu, Wolfgang Gstöttner, Erich Vyskocil

**Affiliations:** Department of Otorhinolaryngology, Head and Neck Surgery, Medical University of Vienna, 1090 Vienna, Austria; faris.brkic@meduniwien.ac.at (F.F.B.); wolf-dieter.baumgartner@meduniwien.ac.at (W.-D.B.); dominik.riss@meduniwien.ac.at (D.R.); thomas.thurner97@gmx.net (T.T.); david.liu@meduniwien.ac.at (D.T.L.); wolfgang.gstoettner@meduniwien.ac.at (W.G.)

**Keywords:** incus vibroplasty, Vibrant Soundbridge, cochlear implantation, progressive hearing loss

## Abstract

Less than 20% of patients with sensorineural hearing loss (HL) provided with the Vibrant Soundbridge (VSB) experience a progressive HL and warrant cochlear implantation (CI). The aim of this study was to identify possible predictors of progressive HL prior to VSB implantation. This retrospective study included all consecutive ears with sensorineural HL provided with the VSB between 1998 and 2016. The patient cohort was divided in a study group comprising patients who underwent CI (CI group) after years of VSB usage and those who did not require VSB replacement during the observational time (control group). Pre- and postoperative pure-tone audiometry thresholds were compared among the two groups. Fifteen out of 81 VSB devices (18.5%) required a CI. The CI group had higher preoperative air-conduction (AC) thresholds than the control group (64.3 ± 8.9 dB vs. 56.3 ± 12.9 dB; *p* = 0.007) at the time of the VSB implantation. On average, the CI group was significantly younger (39.1 ± 12.3 years vs. 52.6 ± 16.2 years; *p* = 0.003). In conclusion, VSB users with higher preoperative AC thresholds and younger age at the time of VSB implantation might be at risk for progressive HL within the upcoming eight years and need a further CI surgery. Preoperative counseling is particularly advisable in this patient group.

## 1. Introduction

The Vibrant Soundbridge (VSB) (MED-EL, Innsbruck, Austria) is an active middle ear implant used for hearing rehabilitation for almost three decades. The indications expanded from sensorineural hearing loss (SNHL) [1,2] to mixed/conductive hearing loss [3,4,5,6]. This advancement was facilitated by implementing the coupling of the floating mass transducer (FMT) to the round window [3,4], oval window [5], and the promontory bone [6]. The short process of the incus (SPI) Vibroplasty was made possible thanks to the new SP-Coupler [7].

Even though the VSB provides stable and effective long-term hearing outcomes [2,4,8], some patients might require a cochlear implant (CI) in the follow-up period. A previous study [2], which included all patients implanted with the VSB, reported that 13 out of 118 ears (11%) required the CI due to progressive hearing loss. To date, there are no articles systematically reporting on patients requiring the CI after VSB implantation. Furthermore, no studies assessed prognostic markers predicting the need for CI after VSB implantation due to progressive hearing loss. Therefore, the aim of this study was to evaluate the number of CI implantations following incus vibroplasty in patients who developed progressive SNHL. Additionally, we aimed to identify prognostic markers that might indicate a risk for a consecutive CI implantation after Incus-vibroplasty.

## 2. Materials and Methods

### 2.1. Patients

In this retrospective cohort study, we analyzed patients who underwent VSB (MED-EL, Innsbruck, Austria) implantation with incus coupling for SNHL prior to being implanted with a CI (MED-EL, Innsbruck, Austria) during the follow-up due to progredient hearing loss and consequent functional insufficient hearing amplification provided by the VSB. The control group comprised all SNHL patients implanted with the VSB who did not need a CI implantation in the follow-up. All VSB implantations were performed at the Department of Otorhinolaryngology, Head and Neck Surgery of the Medical University of Vienna between 1998 and 2016. Clinical data were retrieved using patient medical data records.

### 2.2. Surgery

All VSB devices were implanted by two experienced coauthors (W-D.B. and W.G.). Patients were implanted if they suffered from a moderate to moderately severe SNHL (i) and did not benefit from the hearing aid, (ii) had recurrent external ear infections, or (iii) presented anatomical considerations/narrow external auditory canal that prevented the use of conventional hearing aids. Preoperatively, each patient’s temporal bone was scanned with computer tomography. In patients with SNHL, AC thresholds play a pivotal role in indicating the VSB implantation. The exact indication area for AC thresholds is noted by the implant manufacturer [9].

### 2.3. Audiometric Testing

We compared preoperative hearing thresholds prior to VSB implantation in sound-field (SF) pure-tone audiometry (PTA) of the CI group with the control group. For the analysis, the last available preoperative SF PTA was used. Bone- and air-conduction thresholds throughout frequencies (500, 1000, 2000, 3000, and 4000 Hz) were used for the analysis. In order to cover the contralateral ear, earmuffs were used (Peltor Optime III: 3M, St. Paul, MN, USA). The functional hearing gain (FHG) was calculated by subtracting the unaided by VSB-aided PTA thresholds. The speech recognition in quiet was assessed at 65 Decibel Sound pressure Level (dB SPL) using the Freiburger monosyllabic word test. For the VSB-aided and postoperative unaided PTA thresholds, as well as for the speech recognition in quiet measurements, the last available measurements of the 6-month post-implantation period were used.

### 2.4. Statistical Analysis

We used the Statistical Program of Social Sciences (SPSS Version 23.0, IBM Corp., Armonk, NY, USA) for the statistical analyses. The normal distribution of data was shown by the Shapiro–Wilk Test. Therefore, descriptive results were presented by mean and standard deviations. In order to compare AC-thresholds, FHG, and age between the study group and controls, an independent t-test was used. Unaided and aided AC-Thresholds (FHG) among the same patient groups were compared using the paired *t*-test. The level of statistical significance was set at 0.05, two-tailed. All figures were generated using the Microsoft Excel 2016 (Microsoft Corporation. 2016. Microsoft Excel. Redmond, Washington, DC, USA) and GraphPad Prism (version 9.1.0 for Windows, GraphPad Software, San Diego, CA, USA).

## 3. Results

### 3.1. Patients

Fifteen out of the total 81 VSB devices (18.5%) implanted during the study period were replaced by a CI due to a progressive hearing loss. The CI group comprised 13 patients, with two being implanted bilaterally with the VSB (*n* = 15 devices). The average age at the time of VSB implantation in the CI group was 39.1 ± 12.3 years, while the mean age of the control group (total of 66 implantations, 58 patients, eight bilaterally implanted) was 52.6 ± 16.2 years (*p* = 0.003). In the total cohort, 37 implantations (45.7%) were performed in female patients. For the entire cohort, the mean follow-up was 8.9 ± 5.6 years. In the study group, the average follow-up and the mean time to CI were 12.5 ± 5.3 years and 8.4 ± 5.2 years, respectively. Notably, the preoperative computer tomography temporal bone scans did not show signs of any middle ear or cochlear pathologies. Table 1 shows the demographic data for all VSB implantations, stratified for CI and control groups.

### 3.2. Audiological Outcomes

**Figure 1:**
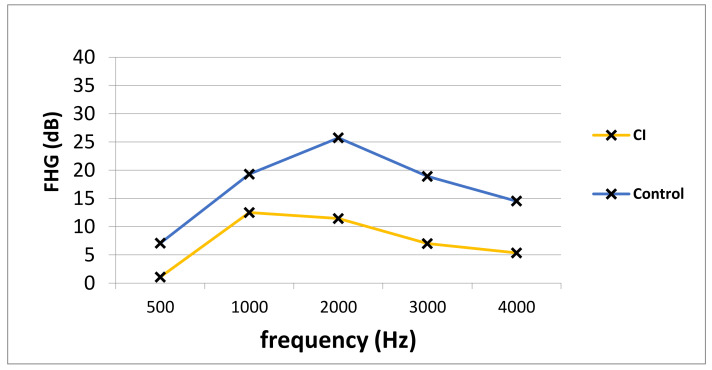
The PTA FHG in the CI and the control group. PTA; pure-tone audiometry, FHG; functional hearing gain, Hz; Hertz, dB; Decibel, CI; cochlear implant group, Control; control group.

**Figure 2:**
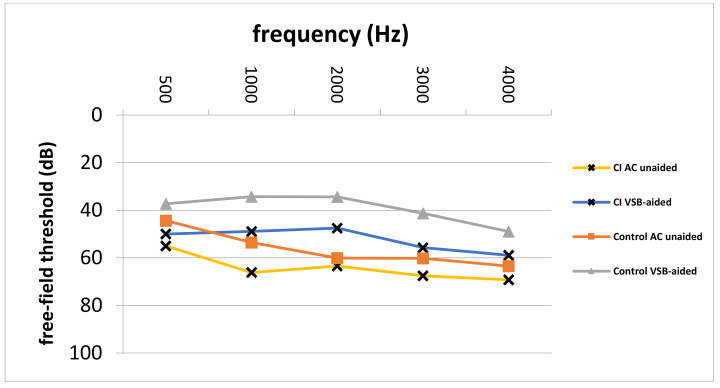
Postoperative unaided AC and VSB-aided thresholds in the CI and control group. CI; cochlear implant, AC; air-conduction, VSB; Vibrant Soundbridge, Hz; Hertz, dB; Decibel, Control; control group.

**Figure 3:**
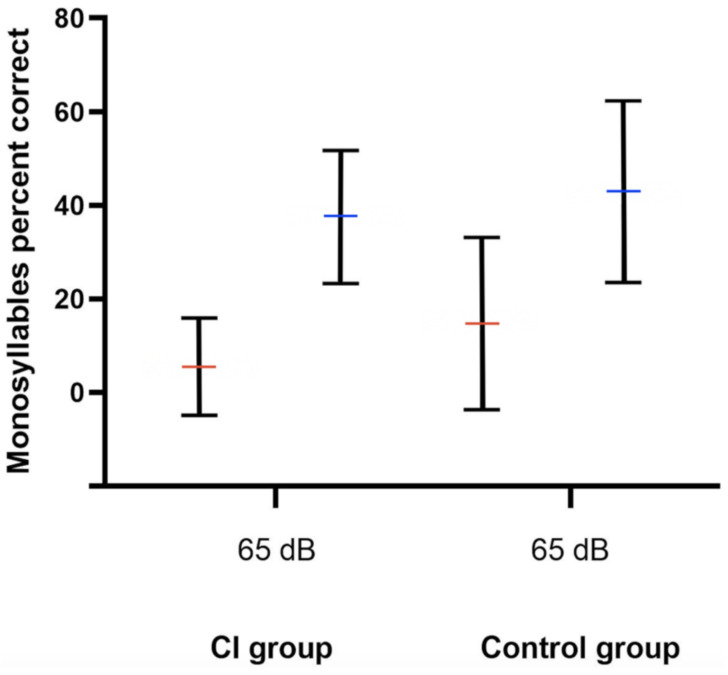
Speech recognition improvement at 65 dB SPL as measured by the Freiburger monosyllabic word test. Red and blue horizontal lines represent the mean unaided and VSB-aided speech recognition, respectively. The whiskers depict the standard deviations. The CI group improved on average from 5.5 ± 10.4% to 37.5 ± 14.2% and the control group from 14.7 ± 18.4 to 42.9 ± 19.4% (*p* = 0.461). dB; Decibel, SPL; sound pressure level, CI; cochlear implant, Control; control group.

Pre- and postoperative PTA, as well as VSB-aided thresholds, were available in a total of 77 out of 81 (95.1%) implantations (14 in the CI group and 63 in the control group). The average PTA from 500 to 4000 kHz in the whole cohort improved from 57.0 ± 12.7 unaided to 41.1 ± 14.7 dB aided, resulting in an FHG of 15.9 ± 18.4 dB. The mean FHG In the CI group was 7.5 ± 29.3. In the control group, the average FHG was higher (17.1 ± 13.9 dB). Nevertheless, the difference in the FHG between CI and the control group was not statistically significantly different (*p* = 0.068), which could be caused by a small patient cohort. Figure 1 depicts the FHG across frequencies for the CI and the control group. Figure 2 shows postoperative unaided and VSB-aided thresholds in the PTA for both groups. In regard to speech recognition, the results of the monosyllabic word test were available for 65 implantations (11 in the CI and 55 in the control group). Overall, the speech recognition improved on average from 13.3 ± 17.7% to 42.1 ± 18.8% at 65 dB, resulting in an improvement of 28.8 ± 15.0%. The CI group improved on average from 5.5 ± 10.4% to 37.5 ± 14.2% (improvement of 32.0 ± 18.1%) and the control group from 14.7 ± 18.4 to 42.9 ± 19.4% (improvement of 28.2 ± 14.3%) at 65 dB. The difference between groups was not statistically significant (*p* = 0.461). This is graphically illustrated in Figure 3. Furthermore, no clinically relevant drop in the BC thresholds was observed in the postoperative PTA (<5 dB at each frequency, on average).

### 3.3. Preoperative Thresholds

**Figure 4:**
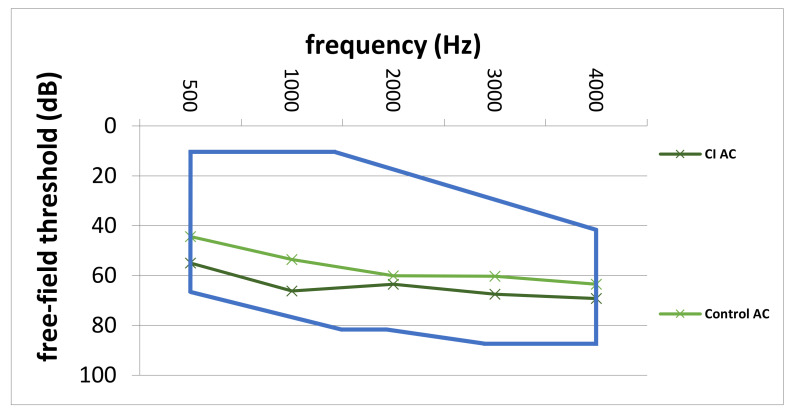
Average pre-VSB AC thresholds across frequencies for the CI and control group. The blue line represents the indication area for AC-thresholds (in the shown frequencies and sound levels) for VSB implantation for patients with SNHL. AC; air-conduction, Hz; Hertz, dB; Decibel, CI; cochlear implant, Control; control group, VSB; Vibrant Soundbridge, SNHL; sensorineural hearing loss.

**Figure 5:**
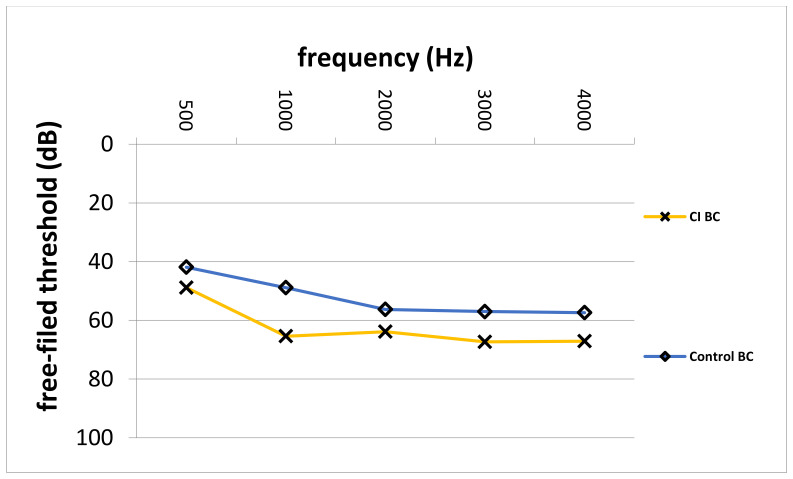
Average pre-VSB BC thresholds across frequencies for the CI and control group. BC; bone-conduction, Hz; Hertz, dB; Decibel, CI; cochlear implant, Control; control group, VSB; Vibrant Soundbridge.

**Figure 6:**
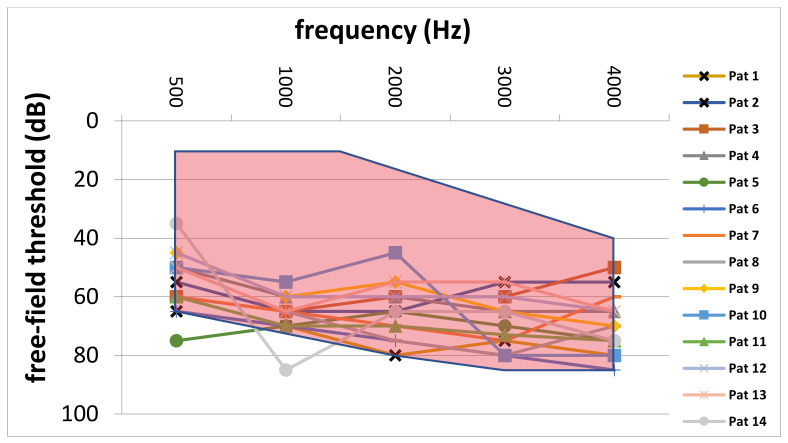
Individual preoperative PTA AC thresholds of patients in the CI group. The red area marks the indication area for AC thresholds (in the shown frequencies and sound levels) for VSB implantation for patients with SNHL. PTA, pure-tone audiometry; AC, air-conduction; CI, cochlear implant; VSB, Vibrant Soundbridge; dB, Decibel; Hz, Hertz; Pat, patient; SNHL, sensorineural hearing loss.

Comparison of preoperative average AC thresholds across frequencies revealed significantly higher values in the CI group (64.3 ± 8.9 dB vs. 56.3 ± 12.9 dB; *p* = 0.007). In particular, on average, preoperative AC values were 8.0 dB higher in the CI group. Figure 4 depicts the comparison of preoperative AC thresholds between the study and control group from 500 to 4000 Hz. Furthermore, the blue line represents the indication area (for shown sound levels and frequencies) as recommended by the implant manufacturer. Furthermore, Figure 5 shows the average pre-VSB BC thresholds for both groups. In particular, average BC values were higher in the CI group (62.5 ± 12.2 vs. 52.3 ± 11.9). Moreover, Figure 6 depicts pre-VSB PTA AC thresholds for individual 14 ears in the CI group (with available PTA values). As shown, the preoperative AC thresholds in 2 out of 14 implantations were out of the indication area at one frequency each and most of the frequencies were in the higher parts of the indication area in all 14 patients.

## 4. Discussion

The aim of this study was to analyze all VSB users implanted for SNHL via incus vibroplasty who, due to progressive hearing loss in the follow-up period, became affected from profound hearing loss and became CI candidates, receiving the later implant upon VSB explanation. The VSB device was initially intended for the treatment of patients with moderate-to-severe SNHL who were unable to use or were dissatisfied with a conventional hearing aid. The VSB closed the treatment gap to CI for patients who could not benefit from conventional hearing aids and showed high satisfaction rates [2,4,5]. We evaluated the incidence of CI after incus vibroplasty and analyzed audiological hearing thresholds prior to VSB implantation in comparison to controls. The study showed that in 15 patients (18.5%) who required the CI after incus vibroplasty, the VSB device was used for an average of 8.4 ± 5.2 years. Furthermore, we observed significantly higher PTA AC thresholds prior to VSB implantation in the CI group. Notably, in most of these ears, the pre-VSB AC thresholds were in the upper part of the indication range.

The excellent results of VSB implantation are already reported in numerous publications. The average reported FHG in the literature ranged from 13.1 to 28.1 dB [1,10,11,12,13]. The average FHG of our cohort in the current study was comparable to results in the literature.

According to the implant manufacturer [9], audiological criteria for VSB implantation in patients with SNHL depend on the preoperative AC thresholds. As shown in Figure 4, the average AC thresholds for each frequency were in the indication range in both patient groups. However, the AC thresholds in the CI group were closer to the top of the indication area. Moreover, the individual preoperative AC thresholds (Figure 6) in most of the 14 patients who required a CI were in the higher range of the indication area. Furthermore, two implanted ears were outside of the indication area in at least one frequency. These results highlight the importance of implanting the VSB devices strictly inside of the indication criteria as recommended by the manufacturer. The analysis also shows what might happen if BC thresholds deteriorate over time, even if AC thresholds are in the indication range at the time of implantation. The VSB is indicated for stable hearing loss, and the outcomes show the importance of identifying progressive hearing loss prior to implantation. 

Interestingly, the mean preoperative AC thresholds in the SF PTA were significantly higher in the group requiring a CI compared to the control group. On average, the differences were statistically significant and about 8 dB higher across frequencies. Furthermore, some of these patients were closer to the upper limit of the indication criteria in the SF PTA. These patients were not only hearing worse than controls at the moment of implantation, but their hearing also deteriorated. The SNHL progression seemingly reached the limits of the VSB after about eight years, on average.

Moreover, the study group was significantly younger than the control group at the time of VSB implantation.

Taken together, these two factors, the higher preoperative AC thresholds in the PTA and the younger age, might indicate a higher risk for CI implantation after VSB vibroplasty in patients with SNHL. The VSB successfully provided good hearing outcomes in the CI group for 8.1 years, on average. It successfully bridged this phase of progressive hearing loss until a CI was necessary due to a lack of benefit.

Our findings might contribute to identifying patients who are at higher risk for progressive hearing loss and who might need a CI after VSB Implantation. Surgeons should particularly assess these patients for progressive hearing loss before indicating surgery. If hearing outcomes are stable, VSB candidates with AC thresholds close to the upper limit of the indication range should be informed of the higher risk for a potential second surgery later in the follow-up. In addition to AC thresholds, we also noted a decreased functional hearing gain in the CI group compared to the control group (*p* = 0.068).

Other underlying factors may cause a progressive hearing loss, such as cholesteatoma [14] or otosclerosis [15]. However, the preoperative temporal bone computer tomography scans (prior to VSB and to CI) did not show any signs of such conditions in our patients. Furthermore, the intraoperative situs in both implantations in all patients in the CI group did not show any signs of cholesteatoma or otosclerosis. Furthermore, ototoxic effects of some drugs (such as cisplatin [16]), as well as exposure to noise [17], can undoubtedly contribute to or cause a progressive SNHL. However, there were no signs of these factors in both patient groups.

The current study faced certain limitations. First of all, this is a retrospective data analysis, and follow-up data could not be retrieved in a few cases; therefore, there is a high risk of selection bias. Moreover, the number of analyzed patients was limited, the number of patients per group and group population were not matched, and therefore some differences at baseline could have possibly been missed (e.g., differences in VSB-aided speech perception or in the FHG between groups). Furthermore, based on this limitation, multivariate analysis and exclusion of confounders could not be performed. However, even in a small cohort, we reported significant differences in preoperative AC thresholds in the PTA.

## 5. Conclusions

This study shows that the VSB provides successful hearing rehabilitation in patients who could not use conventional hearing aids and showed a deterioration of sensorineural hearing in the follow-up. This highlights the importance of careful patient selection and the need to assess for stable hearing loss prior to incus vibroplasty. Candidates with higher AC thresholds and younger age should be specifically assessed before intervention. Counseling patients about the risk for progressive hearing loss is advisable to raise awareness of the likeliness of further surgical intervention. Further studies are warranted for the validation of our results.

## Figures and Tables

**Table 1:** Demographic data of the study cohort.

Patient Group	Mean Age, Years	Male/Female, *n*	Male/Female, %	*n*/%
CI	39.1 ± 12.3	9/6	56.3/43.7	15/18.5
Control	52.6 ± 16.2	35/31	53.0/47.0	66/81.5
All	50.1 ± 16.4	44/37	54.3/45.7	81/100.0

## Data Availability

The underlying data are available from the corresponding author on reasonable request.
